# Tailoring the Molecular Weight of APEG-Based Polycarboxylate Superplasticizers: Mechanistic Insights into the Workability and Compressive Strength of Alkali-Activated Circulating Fluidized Bed Fly Ash Materials

**DOI:** 10.3390/ma18102239

**Published:** 2025-05-12

**Authors:** Xiaojiao Li, Tong Yan, Chuanlong Chen, Xiuchen Qiao, Jin Yuan

**Affiliations:** 1 College of Environment and Ecology, Taiyuan University of Technology, Jinzhong 030600, China; lixiaojiao@tyut.edu.cn (X.L.); 17766216981@163.com (T.Y.); 13695582674@163.com (C.C.); 2 School of Resource and Environmental Engineering, East China University of Science and Technology, Shanghai 200237, China; xiuchenqiao@ecust.edu.cn

**Keywords:** APEG, polycarboxylate superplasticizer, alkali activation, circulating fluidized bed fly ash, workability, compressive strength

## Abstract

This study aims to investigate the effects and mechanisms of polycarboxylate superplasticizers (PCEs) on alkali-activated circulating fluidized bed fly ash (CFBFA) materials. Two PCEs—APEG-500 and APEG-2400—were synthesized using allyl polyethylene glycol ethers (APEG) with molecular weights of 500 and 2400, respectively. Their water-reducing performance and impact on the compressive strength of alkali-activated CFBFA materials were evaluated. The results show that both PCEs exhibited significant water reduction (up to 28% for APEG-2400) in pure CFBFA paste systems, but their efficacy was largely diminished in alkali-activated systems. Compared to the control group without PCEs, APEG-500 improved compressive strength by 20.37% at 1 day and 33.00% at 28 days, while APEG-2400 exhibited lower early strength but achieved a 10.31% strength increase at 28 days. Mechanistic analyses via XRD and FTIR analyses indicated that there was no significant alteration in reaction products, suggesting that the shorter side chains of APEG-500 facilitated particle adsorption and accelerated early hydration. Mercury intrusion porosimetry revealed that PCEs refined the pore structure by increasing harmless pores and reducing harmful ones, with APEG-2400 showing an 11.11% higher proportion of harmful pores compared to APEG-500. SEM observations supported these findings. This study clarifies the relationship between PCE molecular weight and CFBFA material properties, providing a basis for optimizing CFBFA-based cementitious materials.

## 1. Introduction

Global warming, a topic of global concern, has led to a 1.1 °C increase in global surface temperature since the period of 1850–1900, resulting in significant environmental and economic impacts. At 2 °C of warming, there is a high risk of biodiversity loss, with up to 18% of insects, 16% of plants, and 8% of vertebrates likely to lose over half of their geographic range. Economically, climate change could result in global GDP losses ranging from 2.6% to 7.5% by 2100 [1]. Therefore, it is imperative to take urgent action to reduce greenhouse gas emissions. In 2023, the construction and operation of buildings accounted for 34% of global CO_2_ emissions, making this sector the largest contributor to emissions [2]. Alkali-activated materials (AAM) have gained widespread application due to their potential to significantly reduce carbon emissions [3,4]. Circulating fluidized bed fly ash (CFBFA) contains a large amount of amorphous aluminosilicate structures, which are easily chemically activated to participate in reactions, making it an excellent raw material for the preparation of alkali-activated materials [5]. However, the high viscosity of alkali activators and rapid early reactions can result in reduced flowability, thereby negatively impacting the overall workability of the material [6,7]. Considering the superior performance of polycarboxylate superplasticizers (PCEs) in cement concrete, their application in alkali-activated materials has been extensively investigated.

PCEs primarily function by adsorbing anionic groups from their molecular backbone onto the surfaces of material particles and their hydration products, thereby increasing electrostatic repulsion between particles. Simultaneously, the side chains extend into the liquid phase, forming an adsorbed layer on particle surfaces. When these particles approach each other, spatial steric hindrance occurs, and the combined effect of both mechanisms enhances slurry flowability, reduces water demand, and improves workability [8,9,10]. Currently, research is primarily focusing on alkali-activated materials derived from raw materials such as fly ash, slag, and metakaolin, examining their impact on slurry dispersion properties and material strength. However, the results have not been entirely consistent, with experimental outcomes varying widely. To improve dispersion performance and enhance workability, particularly in terms of flowability, sufficient adsorption is a critical factor. The high calcium content in slag facilitates the adsorption of PCEs. Lei et al. [11] synthesized a series of HPEG PCEs with different structures and found that all of them effectively enhanced the dispersion capability in alkali-activated slag. Xie et al. [12] applied PCEs to fly ash samples with varying calcium contents and demonstrated through rheological testing that all samples exhibited increased flowability, with high-calcium fly ash showing superior performance compared to low-calcium fly ash. However, a high calcium content does not guarantee high adsorption. Fan et al. [13] found that while slag can achieve effective adsorption with the anions of superplasticizers, significant competition for calcium ions exists between the superplasticizer and the alkali activator, leading to the desorption of the superplasticizer from the slag surface. Additionally, the molecular design of PCEs significantly influences their adsorption capacity. Superplasticizers with a high anionic character, high molecular weight, and short side chains typically exhibit enhanced adsorption, leading to superior dispersion capabilities [11,14].

PCEs must dissolve without degradation in highly alkaline environments and possess sufficient adsorption capacity to adhere to the surfaces of material particles in order to exert their effects [9]. However, the unique structure of PCEs, which includes functional groups such as carboxyl, ester, and polyether side chains, can lead to decomposition under strong alkaline conditions or result in competitive adsorption with the anions present in alkali activators. This reduces their adsorption capacity and consequently limits their effectiveness. Therefore, the impact of polycarboxylate superplasticizers on the strength of alkali-activated materials remains inconclusive. Jang et al. [15] found that PCEs exert a delaying effect on the setting of alkali-activated fly ash/slag pastes. They observed a positive impact on compressive strength during the first 7 days but acknowledged that this effect may become negative thereafter. Similarly, Alrefaei et al. [16] observed this phenomenon in their study using a single-component Na_2_SiO_3_ as the alkali activator. While the flowability of the alkali-activated material paste containing PCEs was improved, the compressive strength decreased by 7% after 28 days. In contrast, Nematollahi et al. [17] reported that when using a composite alkali activator made from NaOH and Na_2_SiO_3_ solution, the addition of PCEs positively affected workability and increased the slump. However, a significant decrease in compressive strength was observed after 3 days, with reductions reaching up to 29%.

Other studies have indicated that PCEs can improve the porosity of alkali-activated materials, resulting in a denser structure that indirectly affects material strength. For instance, Carabba et al. [18] investigated the effects of adding PCEs under room temperature curing conditions and found improvements in the workability of alkali-activated fly ash geopolymers, although no significant impact on compressive strength was noted. Keulen et al. [19] demonstrated that increasing dosages of PCEs significantly improved both the workability and strength development of alkali-activated fly ash-slag geopolymer concrete. The intrinsic interaction between PCEs and the aqueous environment significantly influenced hydration processes. PCEs interacted with water and material particle surfaces through their carboxyl (-COOH) and hydroxyl (-OH) groups, forming a stable hydration layer. This reduced the diffusion rate of water and ions on particle surfaces, delaying the early hydration process, decreasing both the initial and cumulative heat release, and ultimately affecting the development of microstructure and mechanical properties [20]. Changes in the water environment, such as ionic strength and pH, altered the spatial steric hindrance effect of PCEs, leading to variations in the gradient distribution of nano-mechanical properties within the materials. This microstructural heterogeneity impacted macroscopic strength [21].

Current research presents varying perspectives on the effects of strong alkaline environments on PCEs, with both positive and negative outcomes reported. The differences in raw materials, types and molecular structures of water reducing agents, as well as the types and reaction conditions of alkali activators, all affect the performance of PCEs in alkali activation environments. Currently, regarding the mechanism of action of PCEs in alkali activation materials, it is mostly based on its application mechanism in cement concrete. Although it is known that the functional groups of PCEs are easily affected by strong alkalinity, the exact degradation pathway and the specific mechanism of its effect on water reducing performance and mechanical properties (such as compressive strength) are still unclear. This study synthesized a PCE using allyl polyoxyethylene ether (APEG) with molecular weights of 500 and 2400 to investigate their water-reducing effects. The synthesized superplasticizers were subsequently incorporated into an alkali-activated circulating fluidized bed fly ash system to examine their impact on the compressive strength of the materials, as well as to analyze the underlying mechanisms.

## 2. Materials and Experimental Methods

### 2.1. Raw Materials

In this experimental study, the raw material used was CFBFA, which was obtained from Guofeng Thermal Power Plant, located in Lvliang, Shanxi Province, China. The desulfurization process employed a combination of in-furnace calcium injection and external semi-dry desulfurization methods. The chemical composition of the CFBFA was measured and presented in Table 1.

**Figure 1:**
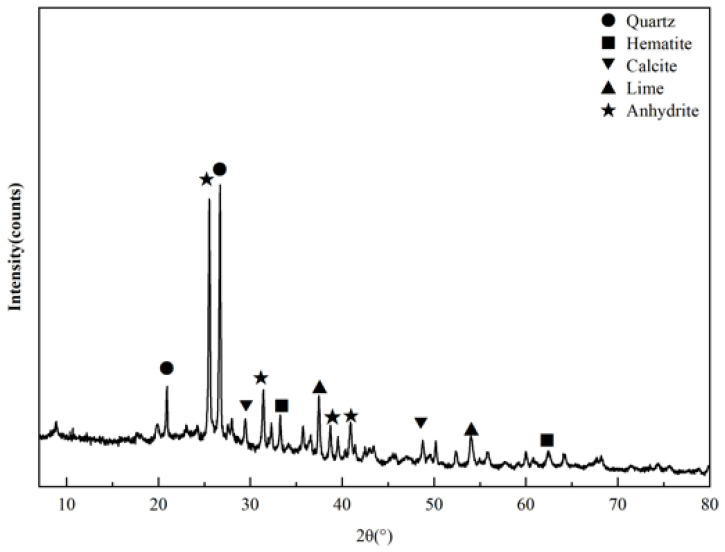
Mineral composition of CFBFA.

The mineralogical analysis of the CFBFA was presented in Figure 1. The primary mineral constituents of the CFBFA included quartz (SiO_2_), hematite (Fe_2_O_3_), anhydrite (CaSO_4_), lime (CaO), and calcite (CaCO_3_).

### 2.2. Preparation of Samples

#### 2.2.1. Preparation of Polycarboxylate Superplasticizers

**Figure 2:**
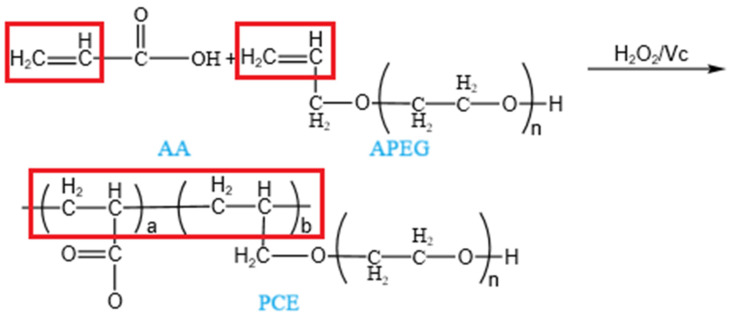
Reaction pathway of polycarboxylate superplasticizers.

In this study, a single-factor method was employed to synthesize the required PCE. During the experiment, allyl polyoxymethylene ether (APEG-500 or APEG-2400) was selected as the macromonomer and placed at the bottom of the reactor. Acrylic acid (AA) served as the small monomer. The initiator consisted of hydrogen peroxide (H_2_O_2_) and ascorbic acid (Vc), while mercaptopropionic acid (MPA) acted as the chain transfer agent. The acid-to-ether ratio was 4, initiator concentration was 1.5%, oxidation-reduction ratio was 2.5, chain transfer agent was 1%, and reaction temperature was 40 °C. The reaction proceeded for 2 h, followed by a 2 h period of thermal insulation. Once the reaction mixture had cooled to room temperature, sodium hydroxide solution was used to adjust the pH to 6–7. The synthesized PCEs were designated as APEG-500 and APEG-2400. The reaction pathway was illustrated in Figure 2. The solid contents of the synthesized APEG-500 and APEG-2400 were 34.05% and 37.52% respectively.

#### 2.2.2. Preparation of Alkali-Activated Materials

NaOH and Na_2_SiO_3_ were selected as composite activators. According to prior experiments conducted by the research group, the compressive strength and performance of alkali-activated CFBFA were optimal when SiO_2_/Al_2_O_3_ = 4.2 and Na_2_O/Al_2_O_3_ = 0.7. Consequently, these parameters were adopted in this experiment. Additionally, considering the standard water requirement for achieving standard consistency of the CFBFA, the water-to-ash ratio was set at 0.7.

1. Preparation of the blank sample: A certain amount of CFBFA and aged activators were added to a mixing bowl and stirred for 2 min to obtain a paste. The paste was then poured into a cylindrical mold (Φ25 × 50 mm) and placed on a vibrating table to eliminate air bubbles. After smoothing the top surface, the mold was transferred to a constant temperature and humidity chamber maintained at 20 ± 2 °C with a relative humidity greater than 90% for curing. After 24 h, the samples were demolded and returned to the curing chamber until the desired age (1 d, 3 d, 7 d, and 28 d) for compressive strength testing.
2. Preparation of samples with PCE: The alkali activator and 2% PCE (by solid content) were thoroughly mixed and added to the mixing bowl along with CFBFA. The mixture was stirred for 2 min to achieve a homogeneous paste. Subsequent steps followed the procedure outlined in step (1).

To prevent further hydration of the samples after compressive strength testing, the samples were placed in a vacuum drying oven and dried at 45 °C to a constant weight. They were then placed in sealed bags, securely sealed, and stored in a desiccator for microstructural characterization.

### 2.3. Analytical Methods

The chemical compositions of CFBFA were analyzed as described in “Methods for chemical analysis of cement” [22]. The solid contents of the PCEs were measured according to GB/T 8077-2012, as described in “Methods for testing uniformity of concrete admixtures” [23].

The workability of the samples was characterized based on the flowability of the paste, which was measured in accordance with GB/T 8077-2012, as described in “Methods for testing uniformity of concrete admixtures” [23].

The compressive strength of the samples was determined according to GB/T 17671-2021, as described in “Test method of cement mortar strength (ISO method)” [24] by using the fully automatic cement flexural and compressive strength testing machine.

The changes in chemical bonds and functional groups of the material were investigated using Fourier Transform Infrared Spectroscopy (FT-IR, PerkinElmer, Waltham, MA, USA). The sample was mixed with KBr at a ratio of 100:1 and pressed into a pellet. The scanning range was from 4000 cm^−1^ to 400 cm^−1^, with a resolution of 4 cm^−1^.

The mineral composition changes of the material were analyzed via X-ray Diffraction (XRD, PANalytical, Almelo, The Netherlands). The working parameters were as follows: 40 kV voltage, 200 mA tube current, Cu target, angular range of 5° to 70° (2θ), scanning rate of 4°/min, and step size of 0.01°.

The pore structure and its variations of the material were analyzed using a high-performance, fully automated mercury intrusion porosimeter (MIP, Micromeritics, Norcross, GA, USA). The bulk sample size was no larger than 1 cm × 1 cm × 1 cm. The mercury intrusion pressure ranged from 0.00069 MPa to 420 MPa, with a fixed contact angle of 130°.

The microscopic morphological changes of the material were analyzed by scanning electron microscope (SEM, HITACHI, Tokyo, Japan), operated at 10 kV.

### 2.4. Statistical Analysis

The compressive strength was calculated as the average of three parallel test results. Data analysis was performed using the SPSS 26.0 software. Given that some datasets did not satisfy homogeneity of variance, a Welch ANOVA was employed to evaluate the statistical significance of reactor performance. Statistical significance levels were defined as follows: * (*p* ≤ 0.05), ** (*p* ≤ 0.01), *** (*p* ≤ 0.001) and **** (*p* ≤ 0.0001).

## 3. Results and Discussion

### 3.1. The Characteristics of Synthesized PCEs

**Figure 3:**
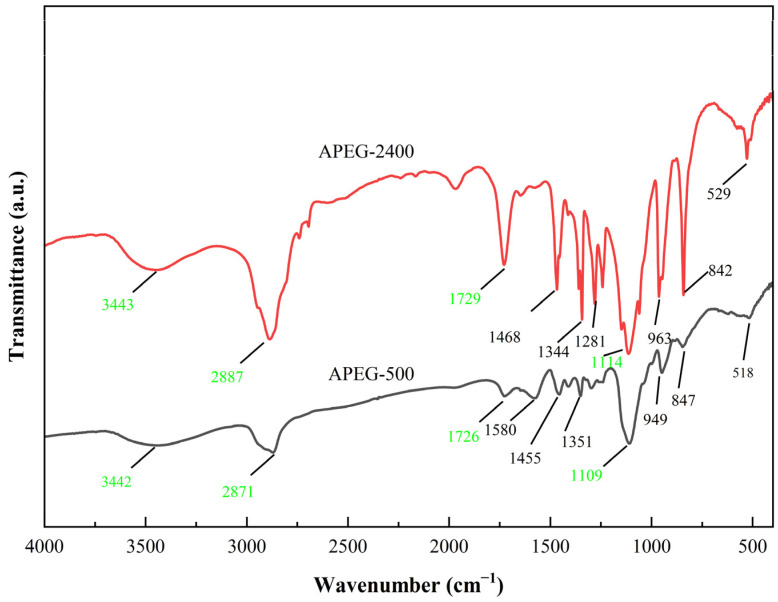
FT-IR spectrum of APEG-500 and APEG-2400.

To confirm the successful synthesis of APEG-500 and APEG-2400 and characterize their molecular structure and chemical composition, FT-IR analysis was used. The FT-IR spectra were shown in Figure 3.

As shown in Figure 3, both the APEG-500 and APEG-2400 curves displayed dense absorption peaks within the wavelength range of 750 to 1500 cm^−1^, corresponding to the stretching vibrations of the C-C main chain. The sharp peaks at 1109 cm^−1^ and 1114 cm^−1^ were attributed to the polyoxymethylene ether characteristic group of C-O-C provided by the APEG macromonomer [25,26]. The characteristic peaks at 2871 cm^−1^ and 2887 cm^−1^ represented the C-H alkyl absorption peak provided by the APEG macromonomer, and the peak for the APEG-2400 was sharper than that of the APEG-500, indicating that APEG-2400 had a longer main chain. A broad peak around 3442 cm^−1^ corresponded to the O-H stretching vibration of hydroxyl groups in the carboxylic acid moiety [25,27]. Additionally, intense absorption peaks at 1726 cm^−1^ and 1729 cm^−1^ indicated the presence of the C=O stretching vibration [25,27,28], confirming the successful grafting of acrylic acid monomers onto the main chain. Analysis of the FT-IR spectrum verified the successful synthesis of PCEs.

By comparing the spectra of APEG-500 and APEG-2400, it was evident that APEG-2400 exhibited more pronounced characteristic peaks with a general shift towards higher wavenumbers. The difference in molecular weight led to distinct chemical environments for the functional groups on the polymer chains, thereby altering the vibration frequencies of chemical bonds and highlighting structural differences [28,29]. APEG-2400, with its higher molecular weight, produced longer polymer chains in the synthesized superplasticizer, resulting in a significantly greater number of side chain groups compared to APEG-500. This increased density of functional groups suggested a higher potential for active sites during interactions with cementitious materials, leading to enhanced adsorption and complexation processes, which in turn had a more substantial impact on the rheological properties of the materials [9].

### 3.2. Effect of PCEs on the Workability of Materials

To investigate the effects of PCEs with different molecular weights (APEG-500 and APEG-2400) on the workability of alkali-activated materials, a series of tests were conducted to evaluate the fluidity of paste under various experimental conditions, as detailed in Table 2. The dosage of the PCE was fixed at 2‰ (solid contents), and the water-to-CFBFA ratio was set at 0.7. The composite alkali activator, consisting of water glass and sodium hydroxide (NaOH), was added according to the calculated ratios of SiO_2_/Al_2_O_3_ = 4.2 and Na_2_O/Al_2_O_3_ = 0.7.

From Table 2, it is evident that both types of PCE significantly enhanced the workability of CFBFA. Specifically, APEG-2400 exhibited superior performance compared to APEG-500. To maintain a flowability of 180 mm for the paste, the water reduction rate of APEG-2400 reached 28%, whereas that of APEG-500 was 23%. This difference can be attributed to the mechanism by which PCEs disperse CFBFA particles through electrostatic repulsion and steric hindrance caused by surface adsorption. The molecular weight of the PCE plays a crucial role in determining the aggregation state of CFBFA particles, thereby influencing their workability. As molecular weight increases, the steric hindrance effect becomes more pronounced [8,9]. From a kinetic perspective, higher molecular weight polymers increase entropy and reduce adsorption free energy, leading to preferential adsorption onto particle surfaces [29]. Additionally, dispersion performance is positively correlated with the thickness of the adsorption layer, which increases with molecular weight. Consequently, high molecular weight PCEs exhibit a more significant dispersion effect on pastes, thereby enhancing their flowability [30].

When PCEs were added to alkali-activated materials, it was found that the pastes exhibited virtually no fluidity and set within about 70 s. This phenomenon could be attributed to the presence of a large amount of calcium-containing phases in CFBFA, which increased the system’s water demand. The CFBFA particles rapidly bound with water molecules from the activator, leading to increased interparticle friction. Simultaneously, the rapid absorption of water molecules reduced the concentration of Na_2_SiO_3_, promoting the formation of silica gel and thereby exacerbating the viscosity of the pastes, which further diminished their flowability. Moreover, the PCEs failed to enhance the flowability of the alkali-activated materials because silicate ions preferentially reacted with calcium ions, preventing the PCEs from effectively adsorbing onto the particle surfaces and thus failing to exert its dispersing effect on the pastes [31,32].

### 3.3. Effect of PCEs on the Compressive Strength of Materials

**Figure 4:**
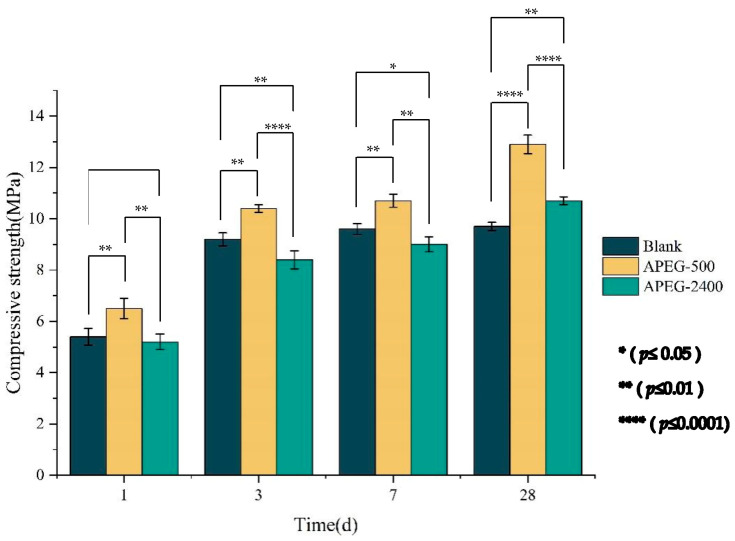
Effect of superplasticizers on the compressive strength of alkali-activated CFBFA materials.

Under the conditions of maintaining a SiO_2_/Al_2_O_3_ ratio of 4.2 and a Na_2_O/Al_2_O_3_ ratio of 0.7, the water-to-binder ratio was fixed at 0.7. The dosages of two superplasticizers were set at 2% (solid content). This study investigated the effect of superplasticizers on the compressive strength of alkali-activated CFBFA materials. The results are shown in Figure 4. As the curing age increased, the compressive strength of all sample groups exhibited an upward trend. However, superplasticizers with different molecular weights had varying impacts on the compressive strength of alkali-activated CFBFA materials. The compressive strength of the samples containing APEG-500 at 1, 3, 7, and 28 days were consistently higher than those of the control group, with increases of 20.37% at 1 day and 33.00% at 28 days. Conversely, the compressive strengths of the samples containing APEG-2400 were lower than the control group at 1, 3, and 7 days, decreasing by 3.70% at 1 day, but showed a 10.31% increase at 28 days. These findings suggest that as the curing age increases, the superplasticizer undergoes changes during the reaction process of the alkali-activated material. Compared with the blank group, samples with APEG-500 showed *p* values below 0.01 at all ages, indicating a significant effect on compressive strength. At 28 days, the *p* value was 0.000004, showing an extremely significant improvement. In contrast, APEG-2400 had no significant effect at 1 day (*p* = 0.65). Although differences increased at 3, 7, and 28 days, improvements were modest compared to APEG-500. Microscopic characterization was employed to analyze these specific changes.

#### 3.3.1. Molecular Structural Changes

**Figure 5:**
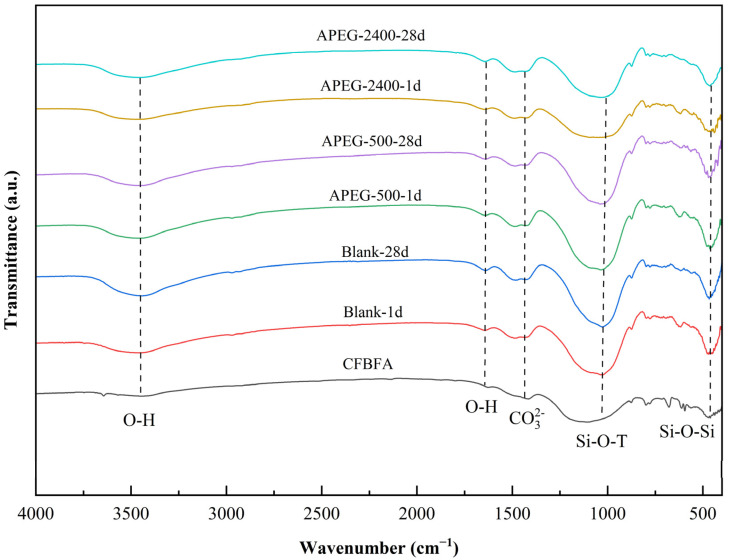
FT-IR spectra of APEG-500 and APEG-2400 on alkali-activated CFBFA materials.

To investigate the effect of superplasticizers on the molecular structure of alkali-activated CFBFA materials, Fourier Transform Infrared Spectroscopy (FT-IR) was employed to analyze the changes in chemical bonds and functional groups, as shown in Figure 5. CFBFA and its alkali-activated samples exhibited a series of distinct characteristic peaks located at approximately 3447, 1640, 1462, 1119, 1010, 979, and 456 cm^−1^. Among these, the absorption peaks at 3447 cm^−1^ and 1640 cm^−1^ primarily corresponded to the bending vibrations of H-O-H bonds, indicating the formation of hydration products in the sample [33]. The absorption peak at 1462 cm^−1^ was associated with the vibrations of CO_3_^2−^ ions [34]. Over time, this peak gradually shifted toward higher wavenumbers, suggesting a gradual shortening of the bond length of CO_3_^2−^ ions. This change in bond length further influenced the compressive strength of the materials. The absorption peak at 456 cm^−1^ represented the bending vibrations of Si-O-Si bonds, reflecting the presence of silicon elements and specific chemical bond structures in the samples. The absorption peaks around 1000 cm^−1^ corresponded to the asymmetric stretching vibrations of Si-O-T (Si, Al) bonds, which were key indicators of the geopolymerization process [35,36]. Compared to raw CFBFA, the absorption peaks of alkali-activated CFBFA, whether with or without the addition of superplasticizers, were generally sharper and exhibited higher intensity. These changes reflected the formation process and structural evolution of the alkali activation reaction.

Comparing the samples before and after the addition of the superplasticizer, it was observed that the addition of the agent had a certain impact on the early reaction process of alkali activation, although the overall effect was not significant. From the 1-day cured samples, compared to the blank group without the superplasticizer, the sample with APEG-500 exhibited an approximately 25% increase in the intensity of the O-H vibration peak, indicating that the hydration reaction was promoted. In contrast, the sample with APEG-2400 showed a slight decrease in the vibration peak intensity, suggesting a mild inhibition of the hydration reaction. Similar trends were observed for the Si-O-T (Si, Al) bonds and Si-O-Si bonds formed during geopolymerization. The C(N)-A-S-H formed by the hydration reaction and the aluminosilicate tetrahedra formed during the geopolymerization reaction, which are the primary contributors to the compressive strength of the samples, also reflected these trends. These findings were consistent with the patterns observed in the compressive strength of the samples. The underlying reason for this phenomenon might have been that APEG-500, with its lower molecular weight and shorter side chains, was more readily adsorbed onto the particle surfaces, resulting in better dispersion and promoting the dissolution of silicon and aluminum. In contrast, the longer chains of APEG-2400 might have caused steric hindrance, thereby affecting the early reaction. However, for the 28-day cured samples, the trends were less evident. This could have been due to the dominance of the adsorption effect of the superplasticizer in the early stages, whereas the later stages involved more complex physicochemical processes. Consequently, the trends determined solely by the molecular weight and side chain length of the superplasticizer became less pronounced [25,37].

#### 3.3.2. Mineralogical Structural Changes

To further investigate the effect of superplasticizers on the mineralogical structural changes in alkali-activated CFBFA materials, both blank samples and those with added superplasticizers were subjected to X-ray diffraction (XRD) analysis after curing for 1 day and 28 days.

**Figure 6:**
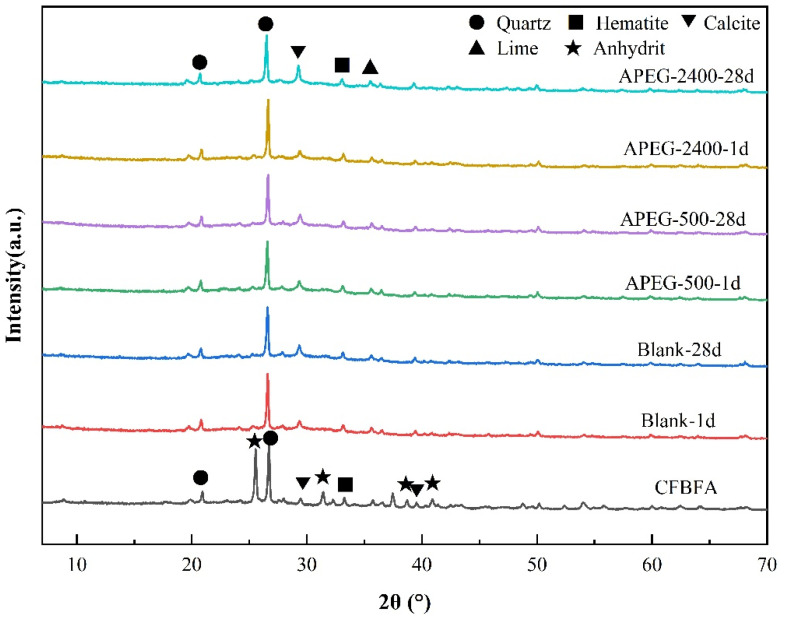
XRD analysis of APEG-500 and APEG-2400 on alkali-activated CFBFA materials.

As shown in Figure 6, when the curing age was only 1 day, the CaSO₄ in the raw CFBFA transformed into a C-A-S-H gel structure, providing the necessary strength for the materials. Notably, the quartz peak intensity of the APEG-500 group decreased more significantly on the first day compared to the Blank and APEG-2400 groups. This indicated that APEG-500 promoted the rapid dissolution of active silica at an early stage, resulting in higher early compressive strength in the samples compared to the other two groups. These findings were consistent with the results from our FT-IR analysis and compressive strength tests of the materials. As the curing age extended from 1 day to 28 days, the quartz peak intensity gradually decreased, indicating that the reaction continued under curing conditions. Active silica and alumina progressively dissolved, and the reaction products continuously increased, leading to sustained improvement in sample strength [33,38]. However, regardless of whether superplasticizers were added, the phase structure of the alkali-activated samples showed no significant changes after 28 days of curing. This phenomenon clearly demonstrated that, despite the incorporation of superplasticizers, they did not alter the phase structure of the reaction products in alkali-activated CFBFA. The alkali-activation reaction continued to proceed, remaining the primary factor responsible for the dissolution of amorphous aluminosilicates in CFBFA.

#### 3.3.3. Pore Structural Changes

**Figure 7:**
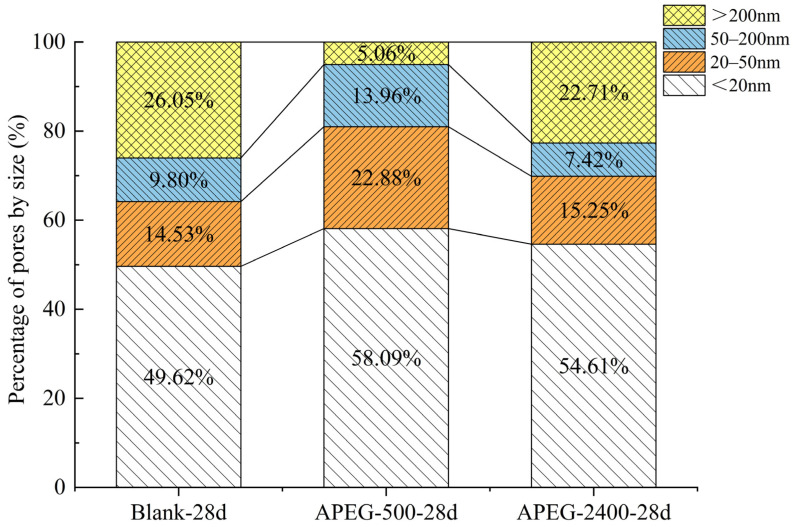
Effects of APEG-500 and APEG-2400 on the proportion of various pore sizes in alkali-activated CFBFA materials cured for 28 days.

**Figure 8:**
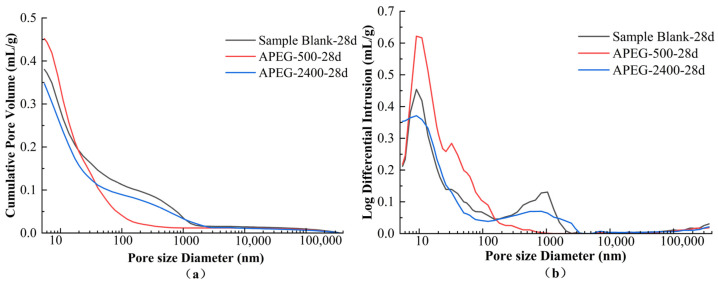
Effects of APEG-500 and APEG-2400 on the cumulative pore volume (**a**) and pore size distribution (**b**) of alkali-activated CFBFA materials cured for 28 days.

To investigate the influence of superplasticizers on the pore structure of alkali-activated CFBFA materials, mercury intrusion porosimetry (MIP) was utilized to examine the porosity and pore size distribution of samples cured for 28 days both before and after the addition of superplasticizers. The results are summarized in Table 3, Figure 7 and Figure 8.

As shown in Table 3, compared to the blank sample, the porosity of the sample with APEG-500 increased, while that of the sample with APEG-2400 decreased; however, the average pore size decreased in both cases. Corresponding to these changes, the compressive strength of all samples containing superplasticizer improved. Therefore, it is not possible to determine the changes in compressive strength solely based on variations in porosity and average pore size. Pores can be classified into four categories based on their size: harmless pores (<20 nm), less harmful pores (20–50 nm), harmful pores (50–200 nm), and more harmful pores (>200 nm). Increasing the proportion of harmless or less harmful pores while reducing the proportion of harmful or more harmful pores is an effective method to enhance strength [39]. As illustrated in Table 3 and Figure 7, the addition of superplasticizers increased the proportion of harmless and less harmful pores while decreasing the proportion of harmful and more harmful pores in alkali-activated materials. This transformation effectively reduced the average pore size by converting larger pores into smaller ones, thereby enhancing pore refinement and filling. Consequently, the microstructure of the geopolymer became denser, leading to improved material strength [40]. Although the workability of PCEs could not be effectively exerted in the alkali-activated CFBFA system, the addition of PCEs could improve the dispersion of the system, making the particles more uniform. During the setting process, this improvement influenced the formation and distribution of pores, thereby potentially affecting the material’s microstructure and mechanical properties [41,42,43].

By comparing the samples with APEG-500 and APEG-2400, it can be observed that although the porosity of the sample with APEG-500 increased by 10.22%, most of the newly formed pores (80.97%) were harmless or less harmful pores, which had minimal adverse effects on strength. As shown in Figure 8, the proportion of pores with diameters >100 nm in the sample containing APEG-500 was the highest. In contrast, the sample with APEG-2400 exhibited a significantly higher proportion of more harmful pores, increasing by 17.65% compared to the APEG-500 sample. This difference may be attributed to the longer main chain of the APEG-2400, which could introduce steric hindrance in the slurry, thereby interfering with the polymerization of [AlO_4_] and [SiO_4_] tetrahedra. Consequently, the more harmful pores were not effectively filled, adversely affecting the material’s structure and performance [19,44]. It was also found that porosity did not have a direct relationship with compressive strength, but that the pore structure distribution was directly related to compressive strength, which was consistent with the findings of Sun et al. [45].

#### 3.3.4. Microscopic Morphological Changes

**Figure 9:**
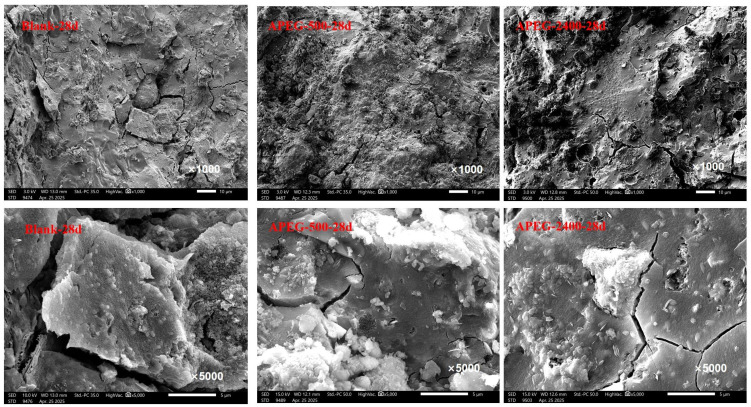
Microscopic morphological changes of alkali-activated CFBFA materials before and after adding superplasticizers.

The microscopic morphological changes of alkali-activated CFBFA materials after 28 days of curing, including the blank sample without superplasticizer, and samples with APEG-500 and APEG-2400, at magnifications of 1000× and 5000×, are shown in Figure 9. The blank samples had numerous irregular cracks, significant surface undulations and protrusions, as well as block-like cracks. At a 5000× magnification, the crack regions displayed pronounced roughness, with many fine particles scattered on the surface, indicating an overall porous and loose structure. This significantly compromised the material’s density and mechanical properties. In contrast, the samples with superplasticizer showed smoother surfaces, fewer cracks, and reduced surface undulations, resulting in a more compact microstructure. Further comparison between the APEG-500 and APEG-2400 admixed samples revealed that the APEG-500 samples exhibited less surface undulation, suggesting a more uniform particle distribution and fewer cracks. Combined with pore structure analysis data, it was evident that APEG-500 effectively increased the proportion of harmless and less harmful pores, thereby enhancing the material’s microstructural density. The microscopic morphological features observed via SEM were consistent with the pore structure analysis results and correlated well with the compressive strength test results.

### 3.4. Application of PCEs in CFBFA-Based Cementitious Materials

The aforementioned research results indicate that APEG-500 and APEG-2400 each possess distinct advantages in water reduction and compressive strength. Specifically, APEG-2400 demonstrates superior water reduction performance when applied to the pure CFBFA slurry system. In contrast, in the alkali-activated CFBFA system, the primary role of water reducers is to refine pore structures and enhance compressive strength, where APEG-500 exhibits greater effectiveness. Regarding the synthesis and selection of PCEs, this study focuses exclusively on APEG macromonomers. However, further investigation into alternative types such as MPEG, HPEG, and IPEG could be beneficial. Additionally, expanding the range of molecular weights considered during selection may lead to improved material properties.

The synthesis, transportation, and application of PCEs must consider environmental impact, safety, and energy consumption. While APEG-500 and APEG-2400 remain stable under standard storage conditions, exposure to strong oxidants during processing must be avoided to prevent adverse reactions. Although their synthesis requires specific energy input, these additives significantly enhance workability and strength, thereby reducing overall energy demands for material transportation and construction. Furthermore, optimizing synthesis temperature and reaction time can further decrease energy consumption in production. Collectively, these factors underscore the potential of PCEs to promote sustainable practices in the construction industry.

## 4. Conclusions

This study investigated the water-reducing effects of polycarboxylate superplasticizers (APEG-500 and APEG-2400) synthesized from APEG macromonomers with molecular weights of 500 and 2400, respectively, and their impacts on the compressive strength of alkali-activated CFBFA materials. The main findings are as follows:

(1) In pure CFBFA paste systems, both superplasticizers demonstrated significant water-reducing effects. APEG-2400 exhibited superior water-reducing performance compared to APEG-500, achieving a 28% water reduction rate when maintaining a fluidity of 180 mm. This enhancement was attributed to the preferential adsorption effect of high-molecular-weight polymers improving particle dispersion. However, in alkali-activated systems, the superplasticizers completely lost functionality, manifested through rapid paste solidification and loss of fluidity. Mechanistic analysis revealed that the preferential reaction between silicate ions in the alkali activator and calcium ions from CFBFA hindered effective adsorption of superplasticizer molecules on particle surfaces.

(2) Compressive strength development displayed notable molecular weight effects. Samples with APEG-500 showed superior enhancement, achieving 20.37% and 33.00% increases in 1 day and 28 days compressive strengths, respectively. In contrast, the APEG-2400 group exhibited a slight 1 day strength reduction (−2.15%) and a 10.31% 28 days strength improvement. This phenomenon originated from the shorter side chains of APEG-500 facilitating early-stage adsorption and dissolution of silicon-aluminum phases, while the long main chains of APEG-2400 created spatial hindrance that inhibited early hydration product formation. Extended curing periods gradually diminished these differences as physical adsorption effects were superseded by complex chemical processes.

(3) Microstructural characterization (FT-IR/XRD) indicated that superplasticizer incorporation did not alter the chemical bonds or phase composition during alkali activation. This confirmed that polycarboxylate superplasticizers primarily function through physical dispersion mechanisms rather than modifying fundamental chemical processes or final reaction products.

(4) Mercury intrusion porosimetry (MIP) analysis revealed that the APEG-500 group showed a 3.2% increase in total porosity, with 80.97% of pores categorized as harmless or less harmful, causing no significant mechanical deterioration. Comparatively, the APEG-2400 group demonstrated 1.8% lower total porosity but contained 11.11 percentage points more harmful pores, correlating with its inferior compressive performance. The microscopic morphological changes observed by SEM were consistent with the situation revealed by MIP. This suggests that the extended main chains of APEG-2400 might interfere with the three-dimensional network formation of [AlO_4_]/[SiO_4_] tetrahedra, impeding effective microporous structure development.

In conclusion, while the current study elucidated the impacts in terms of water-reducing effects and compressive strength of APEG-based polycarboxylate superplasticizers on alkali-activated CFBFA materials, it highlighted the need for further research into the mechanisms affecting PCE functionality in such systems. Future research directions could include exploring strategies to maintain the water-reducing efficacy of PCEs in alkali-activated materials, investigating the effects and mechanisms of different macromonomers such as MPEG, HPEG, and IPEG with varying molecular weights, and studying modified PCEs with grafted comonomers like hydroxyethyl acrylate and sodium methacrylate sulfonate to enhance their performance in alkali-activated systems. These efforts could pave the way for optimizing PCE formulations and advancing the development of alkali-activated cementitious materials.

## Figures and Tables

**Table 1:** Chemical compositions of CFBFA.

Chemical	SiO_2_	Al_2_O_3_	CaO	Fe_2_O_3_	SO_3_	MgO	K_2_O	Na_2_O	LOI
Mass fraction/%	38.85	23.11	15.82	4.94	6.44	1.39	0.31	0.44	3.92

**Table 2:** Test results of the flowability of paste under different experimental conditions.

Tests	Fluidity of Paste (mm)
CFBFA + water	72
CFBFA + water + APEG-500	205
CFBFA + water + APEG-2400	262
CFBFA + water +APEG-500 + composite alkali activator	Set after 72 s
CFBFA + water + APEG-2400 + composite alkali activator	Set after 70 s

**Table 3:** Pore structure changes of alkali-activated CFBFA materials before and after superplasticizer addition.

Sample	Porosity/%	Average Pore Size/nm	Pore Size Distribution/%			
			<20 nm	20–50 nm	50–200 nm	>200 nm
Blank-28d	44.23	17.36	49.62	14.53	9.80	26.05
APEG-500-28d	48.75	15.09	58.09	22.88	13.96	5.06
APEG-2400-28d	42.58	15.67	54.61	15.25	7.42	22.71

## Data Availability

The original contributions presented in this study are included in the article. Further inquiries can be directed to the corresponding author.
